# Corneal Tomographic Changes in Keratoconus Associated with Scleral Lens Wear: A Case-Control Analysis for 12-Month Follow-Up

**DOI:** 10.3390/medicina61040728

**Published:** 2025-04-15

**Authors:** Wei-Hsiang Lin, Tsung-Hsien Tsai, Ching-Hsi Hsiao, Chi-Chin Sun, Jiahn-Shing Lee, Ken-Kuo Lin

**Affiliations:** 1 Department of Ophthalmology, Chang Gung Memorial Hospital, Linkou 33305, Taiwan; marklin1187@gmail.com (W.-H.L.); hsiao.chinghsi@gmail.com (C.-H.H.); leejsh@cgmh.org.tw (J.-S.L.); 2 Department of Ophthalmology, Chang Gung Memorial Hospital, Keelung 20401, Taiwan; drtsaith@gmail.com (T.-H.T.); arvinsun@adm.cgmh.org.tw (C.-C.S.); 3 College of Medicine, Chang Gung University, Taoyuan 33302, Taiwan

**Keywords:** keratoconus, scleral lens, Pentacam HR, tomography, tomographic indices

## Abstract

*Background and Objectives*: Scleral lenses are widely used for visual rehabilitation in keratoconus patients, but their long-term effects on corneal tomography remain unclear. This study aims to evaluate the impact of 12-month scleral lens wear on corneal tomography in keratoconus patients through a case-controlled design. *Materials and Methods*: This retrospective study included 220 keratoconus patients, of whom 10 eyes were treated with SoClear (Brighten Optix Corporation, Taipei, Taiwan) mini-scleral lenses for over one year (SL group). A control group of 14 eyes was matched using Mahalanobis distance matching based on anterior maximum keratometry (K_max_) and age. Both groups were evaluated at baseline and 12 months. Corneal tomography was assessed using the Pentacam HR (Oculus, Wetzlar, Germany), analyzing parameters such as anterior and posterior corneal curvature, thinnest corneal thickness (TCT), and higher-order aberrations. Generalized estimating equations (GEEs) were employed to assess the time-by-treatment effect between the two groups. *Results*: The SL group included 10 eyes from eight patients (seven males, one female; mean age 30.40 ± 6.52 years), while the control group included 14 eyes from 11 patients (three males, wight females; mean age 27.43 ± 8.11 years). Best corrected visual acuity with spectacles improved significantly with scleral lenses (*p* = 0.011) and remained stable (*p* = 0.044) at 12 months. Significant interaction effects were found in Ambrósio relational thickness (*p* = 0.006), posterior radius curvature (*p* = 0.047), posterior mean keratometry (*p* = 0.019), posterior flat keratometry (*p* = 0.023), and thinnest corneal thickness angle (*p* = 0.023); the SL group demonstrated less progression in these parameters compared to the control group. *Conclusions*: This case-controlled study highlights the 12-month impact of scleral lenses on keratoconus, showing improved visual acuity compared to spectacles, stabilized posterior corneal curvature, and maintained corneal thickness. Further prospective studies with larger cohorts are needed to assess scleral lens effect on keratoconus progression.

## 1. Introduction

Keratoconus is a bilateral, asymmetric, non-inflammatory ectatic disorder of the cornea characterized by progressive thinning and steepening, which leads to irregular astigmatism and visual impairment [1]. Typically manifesting in adolescence or early adulthood, the disease may progress over several decades with considerable variability in the rate of progression among individuals [2]. Optical management in keratoconus is particularly challenging with treatment strategies ranging from spectacles and rigid gas permeable (RGP) contact lenses in the early stages to scleral lenses in more advanced cases.

Recently, scleral lenses have emerged as a fundamental component in the management of keratoconus, offering significant optical and therapeutic benefits. As demonstrated by a recent systematic review in 2025, scleral lenses can effectively improve visual acuity and vision-related quality of life, particularly in patients with advanced disease or intolerance to other lens modalities [3,4]. Unlike conventional contact lenses, scleral lenses are designed to vault over the irregular corneal surface and rest on the sclera [5], thereby creating a fluid-filled reservoir that mitigates corneal irregularities and enhances vision. This innovative design renders scleral lenses particularly effective for visual rehabilitation, even in patients with severe keratoconus.

Previous studies have reported that short-term scleral lens wear can cause transient corneal changes, including anterior surface flattening, posterior curvature alterations, and regional thinning [6,7,8,9,10]. These effects were observed in both keratoconus patients with and without intracorneal ring segments or prior cross-linking treatment [8,9,10]. One-year follow-up data showed slight central corneal thickening and vault reduction without limbal stem cell deficiency [11]. Additionally, comparisons among different lens types revealed improvements in visual quality and corneal topography, with scleral lenses showing more stable tomographic parameters [12,13]. However, most of these studies focused on short-term effects or lacked a non-wearing control group, highlighting the need for further controlled investigations. Moreover, the influence of scleral lens wear on the progression of keratoconus remains controversial, with limited studies directly comparing outcomes between scleral lens wearers and non-wearers [14,15]. Therefore, this study aims to evaluate the 12-month effects of scleral lens wear on corneal tomography in keratoconus patients using a controlled study design.

## 2. Materials and Methods

### 2.1. Patient Information

This retrospective study was approved by the Institutional Review Board (IRB number: 202500309B0) of Chang Gung Memorial Hospital (CGMH) and conducted in accordance with the Declaration of Helsinki. We retrospectively reviewed the medical records of patients diagnosed with keratoconus at the Taipei and Keelung branches of CGMH between 1 January 2018 and 31 January 2025.

Inclusion criteria were as follows:

- Patients diagnosed with keratoconus by an ophthalmologist based on clinical signs and corneal topography or tomography;
- Patients with available medical records and follow-up data of at least one year.
- Exclusion criteria wereas follows:
- History of ocular procedures, including intracorneal ring segment implantation, corneal cross-linking, refractive surgery, or cataract surgery;
- Age under 18 years;
- Pregnancy during the study period;
- Presence of ocular diseases other than keratoconus and mild dry-eye disease.

During the study period, a total of 220 patients were diagnosed with keratoconus. Based on the inclusion and exclusion criteria, 8 patients with 10 eyes were treated with SoClear lenses (Brighten Optix Corporation, Taipei, Taiwan)—mini-scleral lenses with a diameter ranging from 14 to 15 mm—for more than one year. These patients formed the scleral lens (SL) group. To establish a case-controlled study with untreated keratoconus patients without contact lenses wear, we performed Mahalanobis distance matching [16] using anterior maximum keratometry (K_max_) with a tolerance of 5 diopters (D) and age, which are the most significant predictive factors of keratoconus progression [17]. Following matching, the control group comprised 11 patients with 14 eyes. Both groups were evaluated at baseline and at 12 months.

### 2.2. Ocular Examinations

All patients underwent a standardized ocular examination at baseline and at 12 months. The protocol included slit-lamp and fundoscopic examinations, as well as measurement of best-corrected visual acuity (BCVA) with spectacles and, for the SL group, BCVA with scleral lenses. Visual acuity was recorded in logarithm of the minimum angle of resolution (logMAR) units. In the SL group, scleral lenses were discontinued for at least one week prior to examination. Corneal tomography was performed using the Pentacam HR (Oculus, Wetzlar, Germany), with parameters measured including anterior flat keratometry (Fr K1), anterior steep keratometry (Fr K2), anterior mean keratometry (Fr Km), anterior maximum keratometry (K_max_), anterior radius of curvature (ARC), posterior flat keratometry (Bk K1), posterior steep keratometry (Bk K2), posterior mean keratometry (Bk Km), posterior radius of curvature (PRC), thinnest corneal thickness (TCT), maximum Ambrósio relational thickness (ART_max_), average pachymetric progression index (PPI), Belin/Ambrósio enhanced ectasia display “D” value (BAD-D), front elevation, back elevation, anterior Q value, posterior Q value, index of surface variance (ISV), index of height asymmetry (IHA), index of vertical asymmetry (IVA), index of height decentration (IHD), keratoconus index (KI), minimum radius of curvature (R_min_), central keratoconus index (CKI), and the locations of K_max_ and TCT. Additionally, total corneal higher-order aberration (HOA) was measured as the root mean square (RMS) from Zernike analysis (up to the sixth order), including RMS values for total HOAs, spherical aberration (SA), and coma.

### 2.3. Diagnosis of Keratoconus and Definition of Keratoconus Progression

Keratoconus was diagnosed based on the presence of at least one ocular sign—such as a Fleischer ring, Vogt’s striae, corneal stromal thinning, corneal protrusion, or corneal scarring—in conjunction with characteristic corneal tomographic findings [1]. Progression of keratoconus over a 12-month follow-up period was defined by at least one of the following criteria: (1) an increase in K_max_ exceeding 1 diopter (D) and/or (2) a reduction in thinnest corneal thickness (TCT) greater than 10 µm [18].

### 2.4. Keratoconus Cone Location

Among the various measurement techniques, TCT and K_max_ were regarded as the cone apex [19]. The pachymetry center on the Pentacam was designated as the reference point. The x and y coordinates of the TCT and K_max_ locations were recorded relative to this center, and the distances from the pachymetry center to these points were calculated. These coordinates were then converted into angular values (in degrees), with the nasal side of the right eye defined as 0°, the superior as 90°, the temporal as 180°, and the inferior as 270°. These angular measurements were referred to as the TCT angle and K_max_ angle for further analysis. To ensure consistency, data from the left eye were mirror-reflected to align with the right-eye coordinate system, mapping the nasal aspect of the left eye to that of the right eye and the temporal aspect similarly.

### 2.5. Scleral Lens Fitting and Safety

Scleral lenses were fitted in a manner that vaults the corneal apex by approximately 300 µm, thereby creating a tear film reservoir while resting on the paracentral cornea [20,21]. Safety was evaluated by monitoring adverse events, including conjunctivitis, corneal edema, progression of corneal scarring, hydrops, and lens dislodgement.

### 2.6. Statistical Analysis

Statistical analyses were conducted using SPSS (version 26.0; IBM SPSS Inc., Chicago, IL, USA). Categorical variables were presented as the number of eyes (percentage) and compared between groups using the chi-square test. Continuous variables are expressed as mean ± standard deviation. Due to the interdependence between the left and right eyes in our design, we employed generalized estimating equations (GEEs) to evaluate the time by group interaction, as well as the main effects of group and time on continuous variables. For any statistically significant effects, post hoc pairwise comparisons were performed. A *p*-value < 0.05 was considered statistically significant.

## 3. Results

### 3.1. Patient Characteristics

In this study, the SL group comprised 10 eyes from eight patients, while the control group included 14 eyes from eleven patients. Among the keratoconus findings, seven eyes exhibited Vogt’s striae, seven showed Fleischer rings, and six presented with corneal scarring. In the SL group, there were seven male and one female patients (mean age, 30.40 ± 6.52 years), whereas the control group consisted of three male and eight female patients (mean age, 27.43 ± 8.11 years). Patient demographics are listed in Table 1. The patient characteristics including BCVA and tomographic parameters at baseline and 12 months are summarized in Table 2.

### 3.2. Location of TCT and K_max_

**Figure 1:**
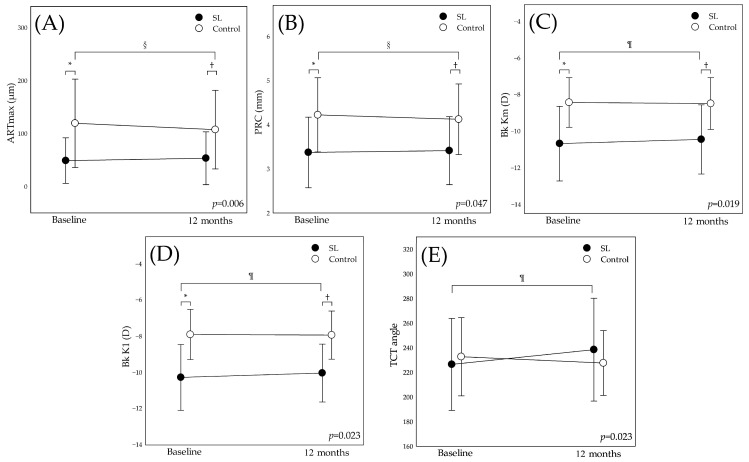
Interaction effects of tomographic indices between SL and control group over 12 months. (**A**) Maximum Ambrósio relational thickness (ART_max_), (**B**) posterior radius curvature (PRC), (**C**) posterior mean keratometry (Bk Km), (**D**) posterior flat keratometry (Bk K1), (**E**) TCT angle. In the figure, the *p* value corresponds to the interaction effect (time by group) derived from the GEE. ¶: significant time change in SL group, *p* < 0.05; §: significant time change in control group, *p* < 0.05; *: significant group difference at baseline, *p* < 0.05; †: significant group difference at 12 months, *p* < 0.05.

In the SL group, the TCT angle and K_max_ angle were 226.41 ± 37.32 degrees and 192.96 ± 82.01 degrees at baseline, respectively, and 238.43 ± 41.81 degrees and 211.44 ± 71.03 degrees at 12 months. In the control group, the TCT angle and K_max_ angle were 232.67 ± 31.76 degrees and 248.38 ± 53.74 degrees at baseline, respectively, and 227.54 ± 26.36 degrees and 245.03 ± 64.23 degrees at 12 months. GEE analysis identified significant interaction effect in the TCT angle (Wald χ^2^ = 5.166, *p* = 0.023), as illustrated in Figure 1. Post hoc analysis of the TCT angle showed no group effect at both baseline (*p* = 0.651) and 12 months (*p* = 0.445). The control group remained stable (*p* = 0.307) though the SL group shifted inferiorly (*p* = 0.033). There was no interaction effect in the K_max_ angle (Wald χ^2^ = 0.917, *p* = 0.338), as illustrated in Supplement S1.

### 3.3. Interaction Effects on Tomographic Indices

GEE analysis identified significant interaction effects for several tomographic indices, including ART_max_ (Wald χ^2^ = 7.509, *p* = 0.006), PRC (Wald χ^2^ = 3.950, *p* = 0.047), Bk Km (Wald χ^2^ = 5.546, *p* = 0.019), and Bk K1 (Wald χ^2^ = 5.173, *p* = 0.023), as illustrated in Figure 1. In contrast, other indices showed no significant interaction effect (see Supplement S1). Post hoc analysis revealed that ART_max_ was significantly higher in the control group at both baseline (*p* = 0.005) and 12 months (*p* = 0.026), with a significant decrease over time in the control group (*p* = 0.007), while remaining stable in the SL group (*p* = 0.263). Conversely, the SL group exhibited significantly greater posterior curvature compared to the control group at baseline (Bk Km: *p* = 0.001; Bk K1: *p* < 0.001) and at 12 months (Bk Km: *p* = 0.004; Bk K1: *p* < 0.001), with these parameters experiencing significant flattening over time (Bk Km: *p* = 0.047; Bk K1: *p* = 0.021), while the control group remained stable with these parameters over time (Bk Km: *p* = 0.171; Bk K1: *p* = 0.608). Additionally, the SL group consistently demonstrated a smaller zonal posterior curvature (PRC) than the control group at both baseline (*p* = 0.009) and 12 months (*p* = 0.021). PRC remained stable in the SL group (*p* = 0.420) but significantly steepened in the control group (*p* = 0.034).

### 3.4. Group Effects on Tomographic Indices

**Figure 2:**
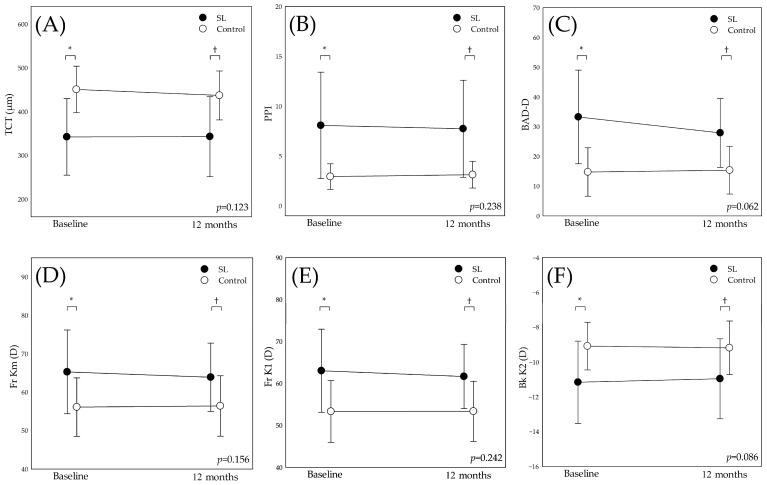
Group effects of tomographic indices between SL and control group at baseline and 12 months. (**A**)Thinnest corneal thickness (TCT), (**B**)average pachymetric progression index (PPI), (**C**) Belin/Ambrósio enhanced ectasia display “D” value (BAD-D), (**D**) anterior mean keratometry (Fr Km), (**E**) anterior flat keratometry (Fr K1), (**F**) anterior steep keratometry (Fr K2). In the figure, the *p* value corresponds to the interaction effect (time by group) derived from the GEE. *: significant group difference at baseline, *p* < 0.05; †: significant group difference at 12 months, *p* < 0.05.

Significant group effects were observed for several tomographic indices. Specifically, TCT (Wald χ*^2^* = 11.465, *p* = 0.001), PPI (Wald χ*^2^* = 9.824, *p* = 0.002), BAD-D (Wald χ*^2^* = 12.738, *p* < 0.001), Fr Km (Wald χ*^2^* = 5.521, *p* = 0.019), Fr K1 (Wald χ*^2^* = 7.974, *p* = 0.005), and Bk K2 (Wald χ*^2^* = 5.940, *p* = 0.015) demonstrated significant differences (Figure 2). Post hoc analysis revealed that at baseline, the SL group exhibited significantly higher values for PPI (*p* = 0.002), BAD-D (*p* < 0.001), Fr Km (*p* = 0.016), and Fr K1 (*p* = 0.006), while the control group had significantly higher TCT (*p* < 0.001) and Bk K2 (*p* = 0.009). These parameters also showed group effects at 12 months.

### 3.5. Impact on Keratoconus Progression

At the 12-month follow-up, keratoconus progression was observed in five eyes (50%) in the SL group and 1ten eyes (71.43%) in the control group, with no statistically significant difference between the two groups (*p* = 0.285).

## 4. Discussion

The strength of our study is that it utilized Mahalanobis distance matching [16] to ensure comparability between groups, with a one-week withdrawal of the scleral lens wear to evaluate its impact on tomographic changes in keratoconus patients for 12-month follow-up. GEE analysis identified significant interaction effects in ART_max_, PRC, Bk Km, Bk K1, and TCT angle between the two groups at the 12-month follow-up. Significant group effects were observed in TCT, PPI, BAD-D, Fr Km, Fr K1, and Bk K2 at baseline and at 12 months. However, BCVA with spectacles showed no significant interaction, group, or time effects between the two groups, indicating that spectacles are not an effective option for managing severe keratoconus.

Variability exists among different devices and methodologies in defining the x and y coordinates of the keratoconus cone location [22,23,24,25,26]. Among the various measurement techniques, TCT on the pachymetry map provided by the Pentacam HR exhibited the highest level of agreement, followed by K_max_ on curvature maps [19]. These findings suggest that TCT may serve as a more reliable reference for cone localization compared to K_max_. Sedaghat et al. found that the keratoconus cone apex was predominantly displaced in the inferotemporal direction on most tomographic maps [19], which is consistent with the findings of our study. Our study also showed that in the SL group, the TCT location shifted inferiorly from the temporal side. This finding aligns with Serramito et al., who reported that short-term (8-h) scleral lens wear resulted in thinning of the inferior cornea [9]. The interaction between scleral lenses, the eyelid, and the tear reservoir warrants further investigation to better understand their combined influence on corneal shape, biomechanics, and physiological responses.

In terms of tomographic parameters, even after matching for K_max_ and age, several indices exhibited significant baseline group differences. Specifically, the SL group exhibited significantly higher values for PPI, BAD-D, Fr Km, and Fr K1, and lower values for TCT and Bk K2, indicating greater initial disease severity compared to the control group. Over the 12-month follow-up, four parameters demonstrated significant interactions effects: ART_max_, PRC, Bk Km, and Bk K1. Our study suggests that scleral lens wear helps preserve ART_max_ and PRC, while also inducing a flattening effect on the BK Km and Bk K1. In contrast, the control group experienced progressive thinning of ART_max_, progressive steepening of PRC, and no significant changes in Bk Km and Bk K1. These findings contribute to a growing body of evidence supporting individualized therapeutic strategies for keratoconus management.

ART_max_, calculated as the ratio of TCT to PPI, has been validated as an accurate parameter for ectasia detection. A steeper increase in corneal thickness from the thinnest point results in a higher PPI [27]. In contrast, PRC represents a posterior radius of curvature within the 3.0 mm optical zone centered on the thinnest point, with higher values indicating a flatter posterior corneal surface [28]. We hypothesize that the preservation observed in the SL group may be related to corneal edema induced by extended scleral lens wear.

Vincent et al. found that scleral lens-induced corneal edema is primarily stromal in nature [29]. Soeters et al. reported that pachymetry significantly increased (*p* < 0.001) immediately after scleral lens removal compared to one week after removal [7]. Fisher et al. further demonstrated that short-term (90-min) open-eye scleral lens wear was associated with central corneal edema, with edema correlating positively with central lens thickness and fluid reservoir thickness. Similar corneal edema increased with the fluid reservoir and was also observed in short-term (90-min) closed-eye scleral lens wear [30,31,32,33]. These findings underscore the potential impact of scleral lens-induced edema on corneal morphology.

Some studies have investigated the impact of scleral lenses on corneal tomography. Kasikci et al. used the Pentacam HR to compare tomographic indices in keratoconus patients over a one-year follow-up among RGP, scleral, and hybrid contact lenses, finding that SL users exhibited more stable changes in TCT along with significant changes in K_max_, pachymetry at the apex, and anterior chamber volume (ACV) [13]. Montalt et al. used the Pentacam HR to evaluate keratoconus patients without a control group for one year follow-up, demonstrating improved BCVA, reduced total HOA, increased contrast sensitivity, greater comfort, and prolonged usage times with scleral lens wear compared to spectacles [34]. There were also a few studies evaluating the short-term effect of scleral lens wear on tomographic changes. Some have reported that short-term scleral lens wear (5 to 8 h) in patients with keratoconus may induce anterior corneal flattening [8,10]. However, Vincent et al. observed a rebound effect, characterized by posterior corneal thinning and flattening, following a 3 h recovery period after lens removal [6]. Iqbal et al. also reported that 6 h of scleral lens wear induced minimal transient changes in anterior corneal curvature and corneal thickness [35].

In our study, scleral lenses in the SL group were discontinued for at least one week prior to examination, allowing the cornea to return to its original state. This washout period minimized any transient effects of scleral lens wear on corneal morphology before assessment. Notably, even after this period, scleral lens wear continued to induce localized posterior corneal flattening while preserving zonal posterior curvature and maintaining corneal thickness stability. These findings suggest a potential influence of scleral lenses on corneal tomography lasting longer than a week, warranting further investigation.

This study has several limitations, primarily its retrospective design and small sample size. The retrospective nature introduces potential selection and information biases—selection bias arises from including scleral lens users with more severe keratoconus, limiting generalizability, while information bias may result from variability in clinical documentation and patient recall. Future prospective studies with larger, well-matched cohorts, extended follow-up periods, or control groups using other RGP lenses are needed to enhance reliability and provide a more comprehensive understanding of the long-term effects of scleral lenses on corneal morphology to refine personalized keratoconus management frameworks and optimize patient outcomes.

## 5. Conclusions

This case-controlled study provides valuable insights into the 12-month impact of scleral lens wear on keratoconus progression and corneal tomography. Despite greater baseline disease severity in the SL group, scleral lens wear led to significant improvement in visual acuity compared to spectacles, stabilization of posterior corneal curvature, and maintenance of corneal thickness without complications. These findings contribute to the advancement of keratoconus management by supporting personalized therapeutic strategies. Further prospective studies with larger cohorts, extended follow-up durations, and appropriate control groups are required to better understand the effects of scleral lens wear on both corneal tomography and keratoconus progression.

## Figures and Tables

**Table 1:** Demographics of study participants.

Characteristics	SL (*n* = 10)	Control (*n* = 14)	*p* Value
Age ^§^, yr	30.40 ± 6.52	27.43 ± 8.11	
<20	1 (10)	0	0.210
20–30	4 (40)	10 (71.4)	
30–40	5 (50)	3 (21.4)	
>40	0	1 (7.2)	
Gender ^§^, male (%)	7 (87.5)	3(27.3)	0.020 *
Severity ^†^ (%)			
grade 2	2 (20)	2 (14.3)	0.711
grade 4	8 (80)	12 (85.7)	
Corneal scarring (%)	7 (70)	1 (7.14)	0.002 *
Fleischer ring (%)	7 (70)	5 (35.7)	0.214
Vogt’s striae (%)	7 (70)	8 (57.1)	0.678

SL: scleral lens; ^§^ the number of age and gender represents the number of cases; ^†^ the severity was evaluated by Amsler–Krumeich classification; * *p* < 0.05.

**Table 2:** Clinical characteristics of study participants.

Variables	Baseline		12-Month Follow-Up		Difference	
	SL (*n* = 10)	Control (*n* = 14)	SL (*n* = 10)	Control (*n* = 14)	SL (*n* = 10)	Control (*n* = 14)
BCVA (logMAR)	0.95 ± 0.67	0.76 ± 0.63	1.12 ± 0.78	0.83 ± 0.56	0.17 ± 0.23	0.07 ± 0.46
TCT (µm)	342.3 ± 87.44	450.71 ± 53.06	342.9 ± 91.33	437.07 ± 55.84	0.6 ± 24.77	−13.64 ± 21.29
K_max_ (D)	77.7 ± 14.28	69.95 ± 11.69	74.97 ± 11.43	70.89 ± 12.35	−2.73 ± 7.41	0.94 ± 1.6
ART_max_ (µm)	48.7 ± 43.06	119.21 ± 83.48	53.2 ± 49.89	107.29 ± 74.29	4.5 ± 13.41	−11.93 ± 17.26
PPI	8.07 ± 5.34	2.94 ± 1.27	7.74 ± 4.87	3.13 ± 1.34	−0.33 ± 1.42	0.19 ± 0.3
BAD-D	33.28 ± 15.74	14.75 ± 8.14	27.9 ± 11.59	15.37 ± 8.03	−5.38 ± 10.62	0.62 ± 1.84
ARC (mm)	5.06 ± 1.01	5.72 ± 0.93	5.09 ± 0.76	5.71 ± 0.87	0.03 ± 0.43	−0.02 ± 0.15
PRC (mm)	3.37 ± 0.8	4.22 ± 0.84	3.41 ± 0.77	4.12 ± 0.8	0.04 ± 0.17	−0.09 ± 0.17
Anterior Q value	−1.51 ± 0.84	−1.39 ± 0.77	−1.50 ± 0.67	−1.41 ± 0.79	0.01 ± 0.32	−0.02 ± 0.1
Posterior Q value	−1.65 ± 0.61	−1.33 ± 0.77	−1.41 ± 0.58	−1.25 ± 0.75	0.23 ± 0.62	0.08 ± 0.38
Fr Km (D)	65.27 ± 10.92	56.09 ± 7.62	63.87 ± 8.92	56.39 ± 7.87	−1.40 ± 3.93	0.3 ± 0.83
Fr K1 (D)	63.01 ± 9.9	53.29 ± 7.33	61.63 ± 7.68	53.33 ± 7.15	−1.38 ± 3.94	0.04 ± 0.98
Fr K2 (D)	67.79 ± 12.22	59.29 ± 8.29	66.36 ± 10.41	59.88 ± 8.94	−1.43 ± 4.22	0.59 ± 2.09
Bk Km (D)	−10.69 ± 2.04	−8.44 ± 1.36	−10.46 ± 1.89	−8.50 ± 1.42	0.23 ± 0.39	−0.06 ± 0.18
Bk K1 (D)	−10.27 ± 1.82	−7.90 ± 1.39	−10.03 ± 1.6	−7.93 ± 1.33	0.24 ± 0.35	−0.03 ± 0.22
Bk K2 (D)	−11.17 ± 2.37	−9.09 ± 1.37	−10.97 ± 2.3	−9.19 ± 1.54	0.2 ± 0.52	−0.10 ± 0.3
Front elevation (µm)	40.7 ± 30.79	39.64 ± 23.33	44.7 ± 21.57	39.5 ± 21.02	4 ± 22.47	−0.14 ± 5.27
Back elevation (µm)	92.16 ± 57.78	79.71 ± 39.05	113.8 ± 39.64	79.86 ± 37.24	21.64 ± 36.81	0.14 ± 11.53
ISV	171.9 ± 60.16	134.71 ± 58.35	163.8 ± 49.06	131.86 ± 58.41	−8.10 ± 27.63	−2.86 ± 10.1
IHA	51.63 ± 42.22	53.7 ± 42.58	40.38 ± 49.65	43.12 ± 45.31	−11.25 ± 19.12	−10.58 ± 31.88
IVA	1.13 ± 0.48	1.09 ± 0.51	1.09 ± 0.48	1.01 ± 0.6	−0.04 ± 0.22	−0.09 ± 0.29
IHD	0.24 ± 0.11	0.21 ± 0.1	0.2 ± 0.08	0.2 ± 0.11	−0.04 ± 0.09	0 ± 0.03
KI	1.43 ± 0.26	1.36 ± 0.2	1.45 ± 0.21	1.35 ± 0.21	0.02 ± 0.13	0 ± 0.06
R_min_ (mm)	4.49 ± 0.9	4.96 ± 0.91	4.6 ± 0.73	4.93 ± 0.94	0.11 ± 0.39	−0.03 ± 0.11
CKI	1.19 ± 0.12	1.17 ± 0.12	1.16 ± 0.09	1.16 ± 0.12	−0.03 ± 0.07	0 ± 0.03
Total HOAs (µm)	2 ± 0.65	1.47 ± 0.72	1.76 ± 0.42	1.54 ± 0.79	−0.24 ± 0.46	0.07 ± 0.52
SA (µm)	−0.79 ± 0.84	−0.72 ± 0.6	−0.61 ± 0.6	−0.73 ± 0.6	0.18 ± −0.01	0.01 ± 0.17
Coma (µm)	1.4 ± 0.5	1.08 ± 0.57	1.25 ± 0.57	1.08 ± 0.72	−0.14 ± 0.63	0 ± 0.52
TCT angle (degree)	226.41 ± 37.32	232.67 ± 31.76	238.43 ± 41.81	227.54 ± 26.36	12.02 ± 18.78	−5.13 ± 19.5
K_max_ angle (degree)	192.96 ± 82.01	248.38 ± 53.74	211.44 ± 71.03	245.03 ± 64.23	18.48 ± 47.34	−3.35 ± 69.27

BCVA: Best-corrected visual acuity of spectacles; LogMAR: logarithm of the minimum angle of resolution; TCT: thinnest corneal thickness; K_max_: anterior maximum keratometry; ART_max_: maximum Ambrósio relational thickness; PPI: average pachymetric progression index; BAD-D: Belin/Ambrósio enhanced ectasia display “D” value; ARC: anterior radius curvature; PRC: posterior radius curvature; Fr Km: anterior mean keratometry; Fr K1: anterior flat keratometry; Fr K2: anterior steep keratometry; Bk Km: posterior mean keratometry; Bk K1: posterior flat keratometry; Bk K2: posterior steep keratometry; ISV: index of surface variance; IHA: index of height asymmetry; IVA: index of vertical asymmetry; IHD: index of height decentration; KI: keratoconus index; R_min_: minimum radius of curvature; CKI: central keratoconus index; HOAs: higher-order aberrations; SA: spherical aberration; SL: scleral lens.

## Data Availability

All data are presented in the article and Supplementary Materials online.

## Supplementary Materials

The following supporting information can be downloaded at https://www.mdpi.com/article/10.3390/medicina61040728/s1, Supplement S1. Generalized Estimating Equations Analysis Results; Supplement S2. Generalized Estimating Equations Analysis Results.

## Abbreviations

The following abbreviations are used in this manuscript:

ART_max_	Maximum Ambrósio relational thickness;
ARC	Anterior radius curvature
BAD-D	Belin/Ambrósio enhanced ectasia display “D” value
BCVA	Best-corrected visual acuity
Bk Km	Back (posterior) mean keratometry
Bk K1	Back (posterior) flat keratometry
Bk K2	Back (posterior) steep keratometry
CKI	Central keratoconus index
Fr Km	Front (anterior) mean keratometry
Fr K1	Front (anterior) flat keratometry
Fr K2	Front (anterior) steep keratometry
HOAs	Higher-order aberrations
IHA	Index of height asymmetry
IHD	Index of height decentration
ISV	Index of surface variance
IVA	Index of vertical asymmetry
KI	Keratoconus index
K_max_	Anterior maximum keratometry
LogMAR	Logarithm of the minimum angle of resolution
PPI	Pachymetric progression index
PRC	Posterior radius curvature
R_min_	Minimum radius of curvature
SA	Spherical aberration
SL	Scleral lens
TCT	Thinnest corneal thickness
