# Supplementary material for: Corneal Tomographic Changes in Keratoconus Associated with Scleral Lens Wear: A Case-Control Analysis for 12-Month Follow-Up
Source: Medicina (Kaunas). 2025 Apr 15;61(4):728. doi: 10.3390/medicina61040728 (PMC12028667; doi:10.3390/medicina61040728)
Supplement: Supplementary file 1 [file medicina-61-00728-s001.zip › Supplement 1_20250413_revised.pdf]

Supplement 1. Generalized Estimating Equations Analysis Results

Tests of Model Effects											
Variable	Wald Chi-Square		p		Wald Chi-Square		p		Wald Chi-Square		p
BCVA	Intercept	50.04	<0.001 *	Fr_Km	Intercept	1162.10	<0.001 *	IVA	Intercept	120.57	<0.001 *
	Group effect	0.89	0.345		Group effect	5.52	0.019 *		Group effect	0.10	0.749
	Time effect	3.15	0.076		Time effect	0.84	0.358		Time effect	1.49	0.222
	Group *	0.54	0.464		Group *	2.02	0.156		Group *	0.23	0.631
TCT	Intercept	691.19	<0.001 *	Fr_K1	Intercept	1313.61	<0.001 *	IHD	Intercept	123.32	<0.001 *
	Group effect	11.46	0.001 *		Group effect	7.97	0.005 *		Group effect	0.16	0.692
	Time effect	1.99	0.158		Time effect	1.24	0.266		Time effect	2.94	0.086
	Group *	2.38	0.123		Group *	1.37	0.242		Group *	2.06	0.151
K_{max}	Intercept	922.00	<0.001 *	Fr_K2	Intercept	998.84	<0.001 *	KI	Intercept	1043.12	<0.001 *
	Group effect	1.50	0.221		Group effect	3.49	0.062		Group effect	0.93	0.336
	Time effect	0.62	0.429		Time effect	0.38	0.540		Time effect	0.18	0.673
	Group *	2.64	0.104		Group *	2.15	0.143		Group *	0.39	0.532
ART_{max}	Intercept	44.89	<0.001 *	Bk_Km	Intercept	767.21	<0.001 *	R_{Min}	Intercept	787.43	<0.001 *
	Group effect	6.46	0.011 *		Group effect	9.39	0.002 *		Group effect	1.41	0.236
	Time effect	1.54	0.215		Time effect	1.76	0.185		Time effect	0.43	0.513
	Group *	7.51	0.006 *		Group *	5.55	0.019 *		Group *	1.38	0.239
PPI	Intercept	49.56	<0.001 *	Bk_K1	Intercept	849.60	<0.001 *	CKI	Intercept	3052.64	<0.001 *
	Group effect	9.82	0.002 *		Group effect	13.01	<0.001 *		Group effect	0.05	0.832
	Time effect	0.10	0.747		Time effect	3.21	0.073		Time effect	1.96	0.162
	Group *	1.39	0.238		Group *	5.17	0.023 *		Group *	1.06	0.304
BAD-D	Intercept	110.04	<0.001 *	Bk_K2	Intercept	653.44	<0.001 *	Total	Intercept	206.36	<0.001 *
	Group effect	12.74	<0.001 *		Group effect	5.94	0.015 *		Group effect	2.49	0.115
	Time effect	2.18	0.140		Time effect	0.33	0.567		Time effect	0.72	0.396
	Group *	3.47	0.062		Group *	2.95	0.086		Group *	2.62	0.105
ARC	Intercept	964.99	<0.001 *	Fr_ele	Intercept	80.12	<0.001 *	SA	Intercept	30.78	<0.001 *
	Group effect	3.44	0.064		Group effect	0.12	0.734		Group effect	0.01	0.931
	Time effect	0.01	0.912		Time effect	0.32	0.575		Time effect	1.13	0.288
	Group *	0.11	0.739		Group *	0.36	0.547		Group *	1.48	0.224
PRC	Intercept	578.99	<0.001 *	Bk_ele	Intercept	117.28	<0.001 *	Coma	Intercept	46.60	<0.001 *
	Group effect	6.15	0.013 *		Group effect	1.89	0.169		Group effect	5.82	0.016 *
	Time effect	0.58	0.446		Time effect	3.63	0.057		Time effect	0.66	0.417
	Group *	3.95	0.047 *		Group *	3.53	0.060		Group *	0.01	0.930
Anterior Q	Intercept	93.94	<0.001 *	ISV	Intercept	189.56	<0.001 *	TCT angle	Intercept	1165.14	<0.001 *
	Group effect	0.11	0.741		Group effect	2.50	0.114		Group effect	0.03	0.864
	Time effect	0.01	0.939		Time effect	1.59	0.207		Time effect	0.83	0.361
	Group *	0.09	0.763		Group *	0.36	0.546		Group *	5.17	0.023 *
Posterior Q	Intercept	134.38	<0.001 *	IHA	Intercept	30.29	<0.001 *	K_{max} angle	Intercept	318.69	<0.001 *
	Group effect	1.01	0.316		Group effect	0.02	0.888		Group effect	3.13	0.077
	Time effect	2.19	0.139		Time effect	4.75	0.029 *		Time effect	0.44	0.507
	Group *	0.56	0.456		Group *	0.00	0.947		Group *	0.92	0.338

BCVA: Best-corrected visual acuity of spectacles; LogMAR: logarithm of the minimum angle of resolution; TCT: thinnest corneal thickness; Kmax: anterior maximum keratometry; ARTmax: maximum Ambrósio relational thickness; PPI: average pachymetric progression index; BAD-D : Belin/Ambrósio enhanced

ectasia display “D” value; ARC: anterior radius curvature; PRC: posterior radius curvature; Fr Km: anterior mean keratometry; Fr K1: anterior flat keratometry; Fr K2: anterior steep keratometry; Bk Km: posterior mean keratometry; Bk K1: posterior flat keratometry; Bk K2: posterior steep keratometry; ISV: index of surface variance; IHA: index of height asymmetry; IVA: index of vertical asymmetry; IHD: index of height decentration; KI: keratoconus index; Rmin: minimum radius of curvature; CKI: central keratoconus index; HOAs: higher-order aberrations; p: p value; SA: spherical aberration; SL: scleral lens

* $p < 0.05$
