# Supplementary material for: Corneal Tomographic Changes in Keratoconus Associated with Scleral Lens Wear: A Case-Control Analysis for 12-Month Follow-Up
Source: Medicina (Kaunas). 2025 Apr 15;61(4):728. doi: 10.3390/medicina61040728 (PMC12028667; doi:10.3390/medicina61040728)
Supplement: Supplementary file 1 [file medicina-61-00728-s001.zip › Supplement 2_20250412.pdf]

Supplement 2 Generalized Estimating Equations Analysis Results

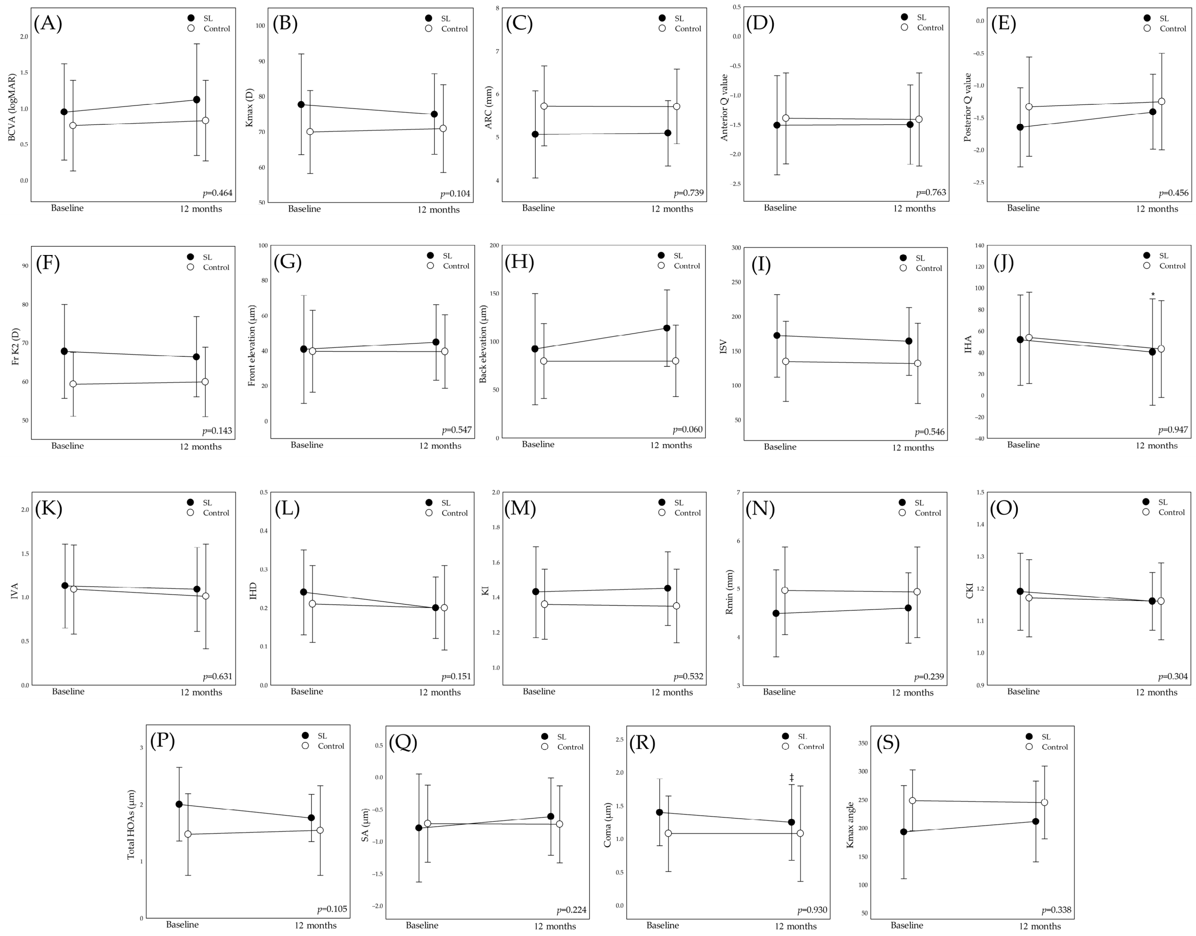

In the figure, the p value corresponds to the interaction effect (time by group) derived from the GEE.
